# Spectrophotometric Analysis of Dental Enamel Staining to Antiseptic and Dietary Agents: In Vitro Study

**DOI:** 10.1155/2020/5429725

**Published:** 2020-06-05

**Authors:** Mukhatar Ahmed Javali, Mohasin Abdul Khader, Razan Mansour Alqahtani, Muna Jubran Almufarrij, Thamra Mohammed Alqahtani, Mohamed Khaled Addas

**Affiliations:** ^1^Division of Periodontics, Department of Periodontics and Community Dental Sciences, College of Dentistry, King Khalid University, Abha, Saudi Arabia; ^2^Intern Dentist, College of Dentistry, King Khalid University, Abha, Saudi Arabia; ^3^Department of Prosthodontics, College of Dentistry, King Khalid University, Abha, Saudi Arabia

## Abstract

**Results:**

The mouthwash containing titanium dioxide (TiO_2_) nanoparticles produced the greater enamel discoloration compared to that of chlorhexidine. Brushing had little effect on removal of stains induced by all mouthwashes except for dietary solutions (lemon with sodium bicarbonate and olive with laurel) and distilled water (control).

**Conclusion:**

The results from this study show that mouthwashes containing TiO_2_ nanoparticles and other antiseptic mouthwashes cause change in color of the teeth and lead to poor esthetic appearance when compared to dietary and control solutions. Thus, future *in vivo* studies have to be conducted to confirm these findings as *in vitro* studies may not provide a reliable simulation of the clinical situations.

## 1. Introduction

Dental caries and periodontal disease are the two common diseases affecting the oral cavity globally [1]. To prevent these oral diseases, many approaches are used out of which mechanical toothbrushing and interdental tooth cleaning are considered the primary and gold standard [2, 3]. However, in some situations or patients such as those affected by trauma or disabled, those undertook oral surgery, those wearing orthodontic braces, and those on chemotherapy, the definite plaque control may not be achieved with mechanical methods [4–7]. In these situations or patients, the use of oral antiseptic in the form of mouthwashes is considered as an adjunct method for controlling dental plaque accumulation and its effect on oral cavity [8, 9].

However, the side effects of these antiseptic mouthwashes such as staining and calculus formation have limited their use for long duration for plaque control. Therefore, finding an alternative antiseptic mouthwash that provides effective plaque control with minimal or no side effects is of great interest [10, 11].

In recent years, the use of nanotechnology in the field of dentistry such as in dental composites and antiseptic solutions is increased [12, 13]. The antibacterial properties of metal ions depend on their surface contact area. Decreased size of nanoparticles (<100 nm in diameter) leads to increased surface area and thus increased interaction with organic and inorganic molecules [12]. Still, many of the properties of metal nanoparticles are still unknown. In addition, discoloration effects and aesthetic changes of some nanoparticles need further explanation.

Conversely, only few studies are available in the literature that has determined the staining effect of these nanoparticles in plaque control. The present study is aimed to analyse the staining effects of antiseptic mouthwashes on dental enamel and compare it with those containing nanoparticles, dietary agents, and distilled water (control).

## 2. Material and Methods

Teeth extracted for orthodontic treatment were collected from the College of Dentistry. 105 premolars were selected for the analysis after excluding teeth with caries, cracks, fillings, or anatomical defects.

The research methodology followed the international standards and ethical directives of the Helsinki. Signed informed consent for study inclusion was obtained from each patient for collection of teeth samples. The study protocol was approved by the Institutional Review Board, and ethical approval was sought from the Scientific Research Committee, the College of Dentistry (SRC/ETH/2018-19/003).

### 2.1. Sample Preparation and Solutions of Staining

Teeth collected for analysis were stored in distilled water at room temperature till the start of the experiment. Before colorimetric analysis, these teeth were cleaned using pumice and rubber cups, rinsed under running water, and then stored in distilled water for 24 hours at room temperature.

After the baseline color examination, R1, the teeth samples were randomly distributed into 7 groups of 15 each. The samples of groups I to VII were immersed in 0.2% chlorhexidine solution (Avohex, Avalon Pharma, Riyadh, KSA), Listerine (Johnson and Johnson, Dubai, UAE), BlanX sodium fluoride 0.05% (Coswell Innovatori Italiani, Italy), lemon with sodium bicarbonate (home-made), olive with laurel (home-made), titanium dioxide nanoparticles (Ultrananotech Private Limited, Bengaluru, India), and distilled water (control), respectively, for a 24-hour period. The samples were then rinsed under running water for a minute, dried with gauze, and colorimetric analysis was repeated, R2 (after immersion in different solutions). These samples were then cleaned using a manual tooth brush (Oral-B, Soft, United States) and tooth paste (Signal, Unilever, United Kingdom) by same examiner using the same technique and position for about 2-3 minutes, dried with gauze, and colorimetric analysis was repeated, R3 (after tooth brushing). For each samples, colorimetric analysis was done thrice during R1, R2, and R3, and the mean of *L*, *a*, and *b* was taken. The color change (Δ*E*) between the readings R1, R2, and R3 was calculated using the following formula:

(1) $$\begin{array}{c} \text{Color change}\left(\Delta E\right)=\left[{\left(\Delta a\right)}^{2}\;+{\left(\Delta b\right)}^{2}\;+{\left(\Delta L\right)}^{2}\right]\times 0.5. \end{array}$$

### 2.2. Preparation of Colloidal Solutions Containing Nanoparticles of Titanium Dioxide (TiO_2_)

TiO_2_ nanoparticles were procured from Ultrananotech Private Limited, Bengaluru, India. The purity of the nanoparticles as per the supplier was more than 99% after ignition. The nanoparticles were added to a water base solution in the research laboratory, the College of Applied Medical Sciences. The colloidal solutions containing nanoparticles were prepared with an initial concentration of 25 ppm and were autoclave sterilized before the experiments. The particle size analysis was performed to assess the size and the distribution of nanoparticles. The average size of the nanoparticles ranged from 40 to 60 nm.

### 2.3. Colorimetric Analysis

For the colorimetric analysis, to assess the color of buccal enamel surface, the Easyshade spectrophotometer (Vita Zahnfabrik, Bad Sackingen, Germany) based on the CIELAB (Commission Internationale de rEclairage *L*^*∗*^ lightness on black white axis, *a*^*∗*^ on red green axis, and *b*^*∗*^ on yellow blue axis) color space system was used [14].

The analysis was carried out at room temperature under natural light. Colorimetric analysis was done following the recommendation of the manufacturer. The Easyshade spectrophotometer device used for colorimetric analysis was calibrated before each color measurement for every tooth.

### 2.4. Statistical Analysis

The data drawn from the study were entered in Microsoft Excel and then checked for normal distribution and the homogeneity of variances by the Kolmogorov–Smirnov test and Levene's test, respectively (*p* < 0.05). One-way ANOVA, analysis of variance, was used to compare the difference in color (∆*E*) between the readings, R1, R2, and R3. Statistical Package for the Social Sciences (SPSS Inc, Chicago, Ill) version 16.0 was used for the statistical analysis, and the significance level was kept at “*p*” value less than 0.05.

## 3. Results

During this *in vitro* study, the analysis process allowed us to gather quantitative data at the 3 stages for each sample. After complete and thorough analysis of the results, Table 1 shows the descriptive statistics and the statistical analysis regarding the difference in color (∆E) between the readings, R1, R2, and R3, among the 7 groups.

**Figure 1:**
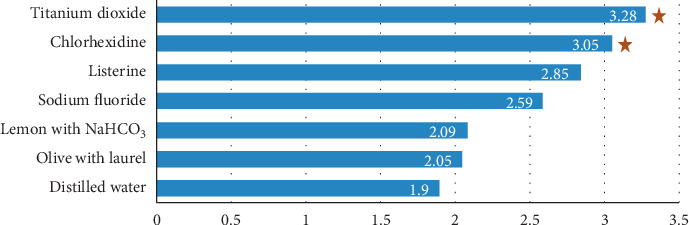
Staining effects of different solutions on enamel (∆*E* R2-R1). ★ Statistically significant (*p* value < 0.05).

Figure 1 demonstrates staining effects of different solutions on enamel (∆*E* R2-R1). Highest difference in color (∆*E*) between the readings R2 to R1 was seen in titanium oxide (3.28) and chlorhexidine (3.05), and the *p* value for the (∆*E*) was 0.001 and 0.021, respectively.

**Figure 2:**
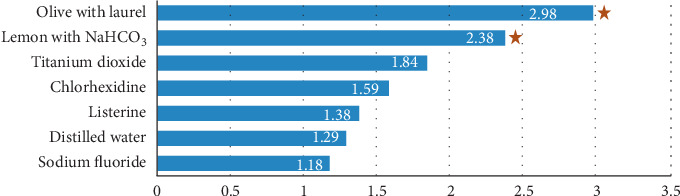
Effect of brushing on stain removal (∆*E* R2-R3). ★ Statistically significant (*p* value < 0.05).

Effect of brushing on stain removal (∆*E* R2-R3) is depicted on Figure 2. Highest difference in color (∆*E*) between the readings R2 to R3 was seen in olive (2.98) and lemon (2.38), and the *p* value for the (∆*E*) was 0.043 and 0.039, respectively.

## 4. Discussion

The success of any treatment modality depends upon biological, mechanical, and aesthetic factors. Aesthetic factors play a major role mainly when the color of the teeth is concerned. The use of antiseptic mouthwashes is recommended as an adjunct to mechanical removal of dental plaque to control caries and periodontal diseases. In addition, use of antiseptic mouthwash is highly advised for the certain situations or patients and during dental implant maintenance.

In this *in vitro* study, the Easyshade spectrophotometer was used for colorimetric analysis of dental enamel. The color of the teeth can be analyzed either using spectrophotometers or colorimeters [15]. These devices lessen the subjective errors of colorimetric analysis with bare eyes, which cannot quantitatively measure minor color changes [16].

This *in vitro* study explored enamel color changes caused by antiseptic mouthwashes on dental enamel and compare it with those containing TiO_2_ nanoparticles, dietary agents, and distilled water. After baseline color measurement (R1), the teeth samples were immersed in different solutions for 24 hours, and color measurement was repeated (R2). A similar immersion period of either 12 or 24 hours was used by Ertas et al. and Celik et al. [17, 18].

Chlorhexidine (CHX) is considered as a gold standard and usually prescribed as a chemical plaque control agent, adjunct to routinely used mechanical removal of dental plaque. It has wide range of activity on bacteria, candida, and viruses [11]. Furthermore, CHX has antibacterial and antiplaque action [10]. However, long-term and frequent use of CHX may have unfavorable effects on oral tissues. Brown staining of teeth, oral tissues, and restorations have been found to be caused by CHX [11]. Staining effect of CHX on enamel was also witnessed in the present study. Most common mechanism to cause brown staining by CHX is evidences because of the interaction or precipitation of anionic dietary chromogens with adsorbed cationic antiseptics [19]. The severity of CHX staining depends on the concentration and the duration of prescription [20].

The third color measurement was done after brushing the teeth (R3). This was performed to analyse the effect of tooth brushing on lessening any stain that has been formed by different solutions. The results of this *in vitro* study showed variable effect on enamel color after immersion in different solutions. The nano TiO_2_-containing solution resulted in the more color change, whereas the staining effects of other solutions were comparable to each other. Tooth brushing had little effect on the removal of stains induced by different solutions, and no significant difference was established regarding the color difference before and after brushing among the groups except for the dietary and control solutions.

On analysis between groups, the results revealed significant differences in color change between R1 (baseline) and R2 (after immersion in mouthwash) stages, as well as between R1 and R3 (after brushing) stage (Table 1). The teeth samples immersed in distilled water and dietary agent solutions explored the least color changes, while CHX and TiO_2_ nanoparticle solutions expressed the higher changes. The difference in color change between the seven solutions was not significant except for CHX and TiO_2_ (Figure 1).

The results of this study showed that staining susceptibility was different for each solution. Results from the study by Silva et al. [21] on artificial teeth showed that food dyes (coffee and red wine) cause higher staining and easily detectable by the human eye compared to CHX. Similar results were shown by Drăghici et al. [22]. However, in this study, the dietary agents showed lesser color changes compared to CHX and TiO_2_ (Figure 1).

When the color change between R1 and R3 was measured, it was found that the teeth samples immersed in nano TiO_2_ solution displayed the higher color changes compared to other solutions and CHX, respectively. This indicates that nano TiO_2_ is not appropriate to be used as oral antiseptic solutions and should be further investigated. It should be noted that the nanoparticle-containing mouthwashes used in this study produced stains with higher than that of CHX; therefore, TiO_2_ nanoparticle-containing antiseptic mouthwash will be not suitable as an alternative to CHX in current available form. Similar results were shown by Eslami et al. [23].

No significant difference was found regarding the color difference before and after brushing among the study groups except for dietary solutions. Brushing had little effect on removal of color changes induced by antiseptic mouthwashes and TiO_2_ nanoparticles (Figure 2). Similar results were shown by Eslami et al. in their study with different nanoparticles [23].

As staining was noticed higher with TiO_2_ in this study, future studies should be conducted to find the exact mechanism of tooth discoloration induced by nanoparticle-containing mouthwashes. However, consideration should also be given to *in vivo* studies to provide a reliable simulation of the clinical situations compared to *in vitro* analysis. The staining effect of different solutions depicting the oral environment and their possible relations should be further explained.

## 5. Conclusion

The mouthwashes containing TiO_2_ nanoparticles produced greater enamel discoloration compared to those of other antiseptic mouthwashes and dietary juices used in the study. Brushing had little effect on removal of stains induced by antiseptic mouthwashes compared to dietary agents and distilled water.

However, within the limitations of the study, that only the teeth extracted because of orthodontic purposes without any age or gender specifications were considered, an *in vitro* study may not mimic the clinical situations, and *in vivo* randomized clinical trials are necessary to establish aforementioned findings, it can be concluded that antiseptic mouthwash on long-term use may cause color changes of the teeth and lead to poor aesthetic appearance. Aesthetic concern of the patients should be taken in consideration when prescribing any antiseptic mouthwash specially those which have found in the literature to affect the color of the tooth enamel. Exploring an alternate antiseptic mouthwash with little or no effect on the color of the teeth to be considered without affecting the antiseptic or antiplaque activity should be taken in future studies.

## Figures and Tables

**Table 1:** Descriptive analysis regarding the difference in color (∆*E*) between the readings, R1, R2, and R3, among the 7 groups.

	Δ*E* (R1-R2)		Δ*E* (R2-R3)		Δ*E* (R1-R3)	
	Mean	SD	Mean	SD	Mean	SD
Chlorhexidine	3.05	1.02	1.59	0.72	3.03	0.98
Listerine	2.85	1.17	1.38	0.85	2.73	1.04
Sodium fluoride	2.59	1.24	1.18	0.68	2.23	0.98
Lemon with NaHCO_3_	2.09	1.09	2.38	0.58	1.97	0.94
Olive with laurel	2.05	1.23	2.98	0.55	1.73	0.88
Titanium dioxide	3.28	1.1	1.84	0.64	3.26	1.0
Distilled water	1.90	1.11	1.29	1.19	1.14	0.72

## Data Availability

The data used for the results of this study are included in the results and discussed in the discussion part of the article.

## References

[1] Ullah R., Zafar M. S. (2015). Oral and dental delivery of fluoride: a review. *Fluoride*.

[2] Saffari F., Ardakani M. D., Zandi H., Heidarzadeh H., Moshafi M. H. (2015). The effects of chlorhexidine and persica mouthwashes on colonization of Streptococcus mutans on fixed orthodontics O-rings. *The Journal of Dentistry*.

[3] Oyetola O., Owotade F., Fatusi O., Olatunji S. (2016). Pattern of presentation and outcome of routine dental interventions in patients with halitosis. *Nigerian Postgraduate Medical Journal*.

[4] Lucchese A., Gherlone E. (2013). Prevalence of white-spot lesions before and during orthodontic treatment with fixed appliances. *The European Journal of Orthodontics*.

[5] Julien K. C., Buschang P. H., Campbell P. M. (2013). Prevalence of white spot lesion formation during orthodontic treatment. *The Angle Orthodontist*.

[6] Benson P. E., Dyer N. P. F., Millett D. T., Furness S., Germain P. (2013). Fluorides for the prevention of early tooth decay (demineralised white lesions) during fixed brace treatment. *Cochrane Database of Systematic Reviews*.

[7] Shahabi M., Ahrari F., Mohamadipour H., Moosavi H. (2014). Microleakage and shear bond strength of orthodontc brackets bonded to hypomineralized enamel following different surface preparations. *Journal of Clinical and Experimental Dentistry*.

[8] Sheen S., Owens J., Addy M. (2001). The effect of toothpaste on the propensity of chlorhexidine and cetyl pyridinium chloride to produce staining in vitro: a possible predictor of inactivation. *Journal of Clinical Periodontology*.

[9] Lamster I. B. (2006). Antimicrobial mouthrinses and the management of periodontal diseases: introduction to the supplement. *The Journal of the American Dental Association*.

[10] Lorenz K., Bruhn G., Heumann C., Netuschil L., Brecx M., Hoffmann T. (2006). Effect of two new chlorhexidine mouthrinses on the development of dental plaque, gingivitis, and discolouration. A randomized, investigator-blind, placebo-controlled, 3-week experimental gingivitis study. *Journal of Clinical Periodontology*.

[11] Zanatta F. B., Antoniazzi R. P., Rösing C. K. (2010). Staining and calculus formation after 0.12% chlorhexidine rinses in plaque-free and plaque covered surfaces: a randomized trial. *Journal of Applied Oral Science*.

[12] Heravi F., Ramezani M., Poosti M., Hosseini M., Shajiei A., Ahrari F. (2013). In vitro cytotoxicity assessment of an orthodontic composite containing titanium-dioxide nanoparticles. *Journal of Dental Research, Dental Clinics, Dental Prospects*.

[13] Sadeghi R., Olia P., Rezvani M. B., Taleghani F., Sharif F. (2010). Comparison of the nanosilver and chlorhexidin antimicrobial effect on Streptococcus sangius and actinomicosis viscosus. *Journal of Islamic Dental Association of Iran*.

[14] Dozic A., Kleverlaan C. J., El-Zohairy A., Feilzer A. J., Khasshayar G. (2007). Performance of five commercially available tooth color-measuring devices. *Journal of Prosthodontics*.

[15] Joiner A. (2004). Tooth colour: a review of the literature. *Journal of Dentistry*.

[16] Jahanbin A., Basafa M., Moazzami M., Basafa B., Eslami N. (2014). Color stability of enamel following different acid etching and color exposure times. *Journal of Dental Research, Dental Clinics, Dental Prospects*.

[17] Ertas E., Güler A. U., Yücel A. Ç., Güler H., Guler E. (2006). Color stability of resin composites after immersion in different drinks. *Dental Materials Journal*.

[18] Celik C., Yuzugullu B., Erkut S., Yamanel K. (2008). Effects of mouth rinses on color stability of resin composites. *European Journal of Dentistry*.

[19] Addy M., Mahdavi S. A., Loyn T. (1995). Dietary staining in vitro by mouthrinses as a comparative measure of antiseptic activity and predictor of staining in vivo. *Journal of Dentistry*.

[20] Hoffmann T., Bruhn G., Richter S., Netuschil L., Brecx M. (2001). Clinical controlled study on plaque and gingivitis reduction under long-term use of low-dose chlorhexidine solutions in a population exhibiting good oral hygiene. *Clinical Oral Investigations*.

[21] da Silva P. M. B., Acosta E. J. T. R., Jacobina M., Pinto L. d. R., Porto V. C. (2011). Effect of repeated immersion solution cycles on the color stability of denture tooth acrylic resins. *Journal of Applied Oral Science*.

[22] Drăghici R., Preoteasa C. T., Popa L. (2017). In vitro spectrophotometric evaluation of acrylic teeth staining related to dietary and oral antiseptic agents. *Farmacia*.

[23] Eslami N., Ahrari F., Rajabi O., Zamani R. (2015). The staining effect of different mouthwashes containing nanoparticles on dental enamel. *Journal of Clinical and Experimental Dentistry*.

